# Ethics of Treatment Decisions for Extremely Premature Newborns with Poor Prognoses: Comparison of Shared Decision Making in Norway and Japan

**DOI:** 10.3390/pediatric14040057

**Published:** 2022-11-03

**Authors:** Akira Akabayashi, Eisuke Nakazawa, Hiroyasu Ino

**Affiliations:** 1 Department of Biomedical Ethics, Faculty of Medicine, University of Tokyo, 7-3-1 Hongo, Bunkyo-ku, Tokyo 113-0033, Japan; 2 Division of Medical Ethics, New York University School of Medicine, 227 East 30th Street, New York, NY 10016, USA

**Keywords:** premature newborn, shared decision making, resource allocation, NICU, ethics, neonatology

## Abstract

Ethical debates about the life-prolonging treatment of extremely premature infants and infants with congenital abnormalities with poor prognoses have long been held. We will examine approaches in Norway and Japan as examples because Norway is a well-known welfare state. By comparing the traditional Norwegian approach, the newly proposed approach of postponed withholding (PPWH) and the Japanese approach, we will revisit shared decision making in neonatology in general, where patients (i.e., newborns) inevitably have no decision-making capacity. We argue that in shared decision making, the process is critical, and that it is important to clarify who will be the final decision-maker and whose benefits are most important. In addition, we argue that the issue of cost cannot be avoided in this current time of economic disparities in global health. Shared decision making should not be a mere formality. These are significant examples of new ethical debates to be discussed in the modern era in the neonatology field.

## 1. Introduction

The Baby Doe case in 1982 spurred many discussions and various activities. Among them, the importance of shared decision making was widely advocated. In addition, the cost of life-prolonging treatment in the NICU continues to be an issue, as this type of treatment is extremely expensive. Based on the Norwegian system, Syltern et al. propose a new approach named postponed withholding (PPWH), which tries to empower parents as competent final decision-makers [1]. The aim of this study is basically to compare Norwegian and Japanese approaches to treatment decisions for extremely premature newborns with poor prognoses as an example. We first focus on who will be the final decision-maker in the shared decision making settings. Another purpose is to explore the meaning of shared decision making in this current time of economic disparities in global health.

## 2. Shared Decision Making

Shared decision making refers to a process in which the people involved in decision making collaborate to make a decision. Those involved in deciding on treatment for a premature infant include parents, relatives, friends of the parents, medical personnel, hospital ethics committees, and others. Our first question is, assuming that the idea of shared decision making is sound, who will be the final decision-maker when various and divergent opinions for treatment arise? Additionally, who will be responsible for the decision (presumably, the final decision-maker)? This point has not been adequately examined.

1. Case Study: Japan

One university hospital divides its treatment plan for critically ill newborns into four categories (Table 1) [2].

Treatment decisions are made through shared decision making. Nishida explained shared decision making as follows [3]:

*“Medical decision-making process: The attending physician and the NICU supervisor lead a discussion among those involved in the medical and nursing care of the patient based on medical and family information, and taking into account baby’s best interests. The NICU director confirms whether there are different opinions on the medical outlook regarding the possibility of recovery and treatment. Based on the results of the discussion among the medical personnel, the NICU supervisor will make a final decision on the level in which to place the child as indicated in the table.”*

*(p. 140)*

*“Medical decision-making in Japan differs substantially from that in Europe and the United States in that, in principle, the family is not pressured to make the final decision. The reason for this is that in Japan, especially in neonatal care, where medical information is scarce and mental preparation is inadequate, the opinions of the medical staff have a large impact on the family’s decision-making, and the medical staff thinks that they should bear the burden of decision-making for the rest of their lives rather than leaving it to the family. However, if a family member has a clear opinion, that opinion naturally takes precedence. In addition, the final decision is made by the NICU director; this differs from Europe and the U.S., where there is a third-party decision-making body such as an ethics committee. In principle, the final decision is made through discussions among all the personnel involved in the medical and nursing care of the patient, with the responsible person representing the opinions of those involved. During the discussion process, if there is a clear difference of opinion among the medical personnel, it is important not to rush to a decision or change the policy without thorough consideration.”*

*(p. 142)*

The statement that the NICU supervisor is the final decision-maker may give a paternalistic impression, but it is followed by the statement, “However, for families who have clear opinions, that opinion naturally takes precedence”, which negates paternalism. Instead, the benefit to the parents (that the burden on them is reduced by the medical provider assuming final responsibility) is given priority.

2. Case Study: Norway

Norway is a Western country that emphasizes self-determination. Recently, Syltern et al. (2021) published an article in the *American Journal of Bioethics* entitled “Postponed withholding (PPWH): Balanced decision-making at the margins of viability” [1]. The authors’ definition of PPWH is quoted verbatim below.

The basic idea of this approach is, firstly, to regard the provision of life support at birth as a *non-decision*. Secondly, after a thorough counseling process within a shared decision-making model, further provision of life support should depend on *active* parental request for continuation. We believe that this change in the NICU choice architecture will contribute to empowering parents and enable them to act based on their situation and values. (p.1)

In Table 2, PPWH is compared with the traditional approach in Norway (originally Table 1 in Syltern’s article).

## 3. Evaluations of Syltern’s Approach

This is indeed shared decision making. The premise of PPWH is that parents have the right to surrogate decision making—namely, the final decision-makers—and that empowering parents promotes parental self-determination. Indeed, empowering parents is an excellent approach, and an approach that seeks parental self-determination to the extreme. Because this is a cultural difference, we will not discuss it here. What we are concerned with is a description of the process of shared decision making.

### 3.1. Psychological Invasion of Parents

Syltern et al. differentiate between PPWH and the “Traditional Approach” in Norway, describing the postponement of levels of life support until parents are sufficiently prepared; memory-making (taking pictures, etc.); and active counseling. However, the parents may have diverse views of life and hold different values, which may lead to conflicts arising between them. Parents facing this situation could have several reactions. Some parents may say, “we want to be left alone, we don’t want to be bothered.” Others may not be able to tolerate the process of memory-making and may wish to “quickly” forget.

Syltern et al. also state that “The intention of PPWH is not to nudge, but to strengthen volitional autonomy.” They use such phrases as “choice architects”, “immediate instinct”, and “withdrawal aversion”. These terms correspond to “choice architects”, which Kahneman (2011) calls “System 1” [4] and “risk aversion”, and seem strongly influenced by behavioral economics, similar to nudge theory and prospect theory. The purpose of selecting PPWH over the other three options is to correct the bias in parental behavior, which might be regarded as a nudge intervention. It is undesirable to disguise PPWH as “unintended”. Rather, if it is based on a nudge framework, the authors should ethically justify their positions in asserting so.

### 3.2. The Illusion of True Parental Intentions—To What Extent Do We Require Final Decision Making by Parents?

It is optimistic to assume that shared decision making can lead to truly autonomous parental expressions of intent. We assume that neither counseling nor spiritual care will help parents become fully competent in making decisions and strengthen their volitional autonomy to enable them to express their true intentions. We speculate that parents in this position may be mostly vulnerable, confused, and want to shelve the issue; many parents would remain incompetent surrogate decision makers.

One concern to parents is the content of “active counseling”. Active counseling could be interpreted as a move toward stopping treatment early. The description “memory-making (taking pictures, etc.)” might be understood in the direction of stopping treatment. Shared decision making, of course, has no objective correct course. However, the question remains: For whom does “shared decision-making” exist? The process of shared decision making in each region and culture must be carefully discussed.

### 3.3. Psychological and Economic Costs—Who Bears the Costs?

We were surprised to see that the “traditional approach” in Norway (Table 2, originally Table 1 in Syltern’s article) includes “emotional and spiritual support”. Do they employ specialized spiritual care and counseling personnel in Norway? Or are nurses, MSWs, and other healthcare workers providing these services? Either way, this type of care is expensive and time-consuming. Japan is said to have a relatively good healthcare system, but with fragile financial conditions. There is no money to hire such personnel in busy NICUs, nor can staff afford the time to provide spiritual care. Besides this, we have to keep in mind that there are many poorer countries that cannot even afford to have NICUs and other expensive facilities.

We surmise that current medical systems around the world do not always provide initiation of life-support at birth for all extremely premature infants. Even if life-support is provided in all cases, invasive procedures such as intubation, intravenous routes in difficult-to-reach blood vessels, and arterial lines performed by the medical staff would prove to be painful for the infants.

Furthermore, medical costs would rapidly increase if the trial of life support as a non-decision is conducted. These costs would have to be borne mostly, if not entirely, by the parents. Such a situation also leads to the ethical issue of micro-level resource allocation: If the scarce NICU beds are occupied, other critically ill neonates, who could otherwise be saved, would not be admitted. Moreover, who would pay for the professional counselors providing active counseling? In Japan, traditionally, we assume that extended family gather and support the parents. Therefore, we believe that expensive active counseling is not needed in Japan, depending on the content of the counseling.

### 3.4. Can We Prevent the Suffering of NICUs in a World Where Even the Traditional Norwegian Approach Is Not Widespread?

In developed countries such as Norway (and probably most European countries), shared decision making has already been used as a traditional approach, and in NICUs, emotional and spiritual support is provided, and attachment is “imperative”. How to allocate limited healthcare resources is a matter of debate relevant to all forms of healthcare, in most societies worldwide. Although its ideals are preferrable, emotional and spiritual support is highly expensive in the context of the global healthcare environment. However, as healthcare is a basic necessity for all people in the world, we should first focus our efforts on promoting the “traditional approach”.

Imbalances and cultural differences in legal and social systems must also be considered. For example, consider the cultural differences in the withdrawal of life support. There is a strong sense of rejection of the withdrawal of life support in Japan. Once a patient is fitted with a ventilator, the act of removing it is considered a legal “commission”, and the possibility of being legally guilty of murder has not yet been ruled out (Nakazawa 2019) [5]. If a respirator were placed with a non-decision in Japan, the patient and the attending physician would face a difficult situation because a respirator cannot be easily removed. Since each country has its own system, life support with a non-decision is not easy to implement according to each country’s legal and social system.

### 3.5. Does Empowering Parents Always Lead to Ethically Justifiable Consequences?

#### Norway’s non-decision proposal immediately reminds us of the case of anencephalic Baby K, born in 1992 in Virginia, USA (Fletcher 1994).

*“Anencephalic children are completely or almost completely devoid of a cerebral cortex congenitally; however, the brainstem is present. Baby K, born with respiratory failure, was placed on a ventilator to confirm the diagnosis, and to allow the mother to say goodbye. Only comfort care was provided to Baby K; the medical staff had decided against providing active care to the baby. To obtain a DNR order, the mother was informed of the patient’s condition. However, the mother, a devout Christian who firmly believed that all life should be protected, insisted that there might be a miracle from God and demanded all possible treatment. Baby K was repeatedly admitted and discharged from the hospital’s ICU. However, the district court and the circuit court ordered the treatment to be continued on the grounds of anti-discriminatory law. The hospital referred the case to the Supreme Court; however, the case was rejected. Baby K is said to have survived for more than three years.”*

[6]

Empowering parents in this way may be desirable, but if these empowered parents demand endless treatment that is considered futile by healthcare providers, the psychological costs to healthcare providers and the economic costs of treatment will be enormous.

## 4. Conclusions

Shared decision making has been revisited herein. Although the self-determination rights of the surrogate parents are important, the best interests of the infants and parents are equally important. Limitations include the fact that this discussion is a comparison of only two countries, and universality is not guaranteed, though we believe this discussion would be useful to other countries. In this paper, we do not discuss economic disparities, since this is beyond the scope of this paper. However, it is necessary for people in each country and region to think locally as well as globally. We also have to work towards improving access to neonatal care in the developing world, and not be limited to the developed one. We hope that this article will help to ensure that shared decision making will not become a mere formality, but will, in fact, be beneficial to all those involved, especially in neonatal care settings. These are significant examples of ethical debates to be discussed in the modern era of the neonatology field, wherein patients do not ever have decision-making capacities.

## Figures and Tables

**Table 1:** Post-decision medical policy.

Class A: The patient would be given all types of treatment. This is mostly for child patients. Class B: No treatment is provided beyond a certain limit (cardiac surgery, hemodialysis, etc.). This is for patients with a clear poor prognosis such as epidermolysis bullosa or congenital myopathy. Class C: General care (warmth, nutrition, wipes and affection) is given without any further treatment. Examples of such patients are those with 13-trisomy, 18-trisomy or anencephaly; very premature infants under 500 g born in severe distress; neurologically unresponsive infants with severe intracranial hemorrhage during ventilation, etc. Class D: No treatment is given.

**Table 2:** Core elements of PPWH compared to the traditional approach.

	Traditional Approach	Postponed Withholding (PPWH)
**Threatening** **birth in defined gray zone**	Information Shared decision making: Prenatal steroids, transfer Mode of delivery Life support or comfort care at birth	Information Shared decision making: Prenatal steroids, transfer Mode of delivery Level of life support postponed until parents are sufficiently prepared
**At birth**	Decision at the discretion of attending neonatologist, based on best interest of infant and informed by parental wishes	Trial of life support as a **non-decision** (should not surpass “harm threshold”); comfort care possible if there is a proper prenatal shared decision-making process
**In the NICU**	Parental participation in care Decision making at the discretion of the neonatologist; emotional and spiritual support; “Attachment imperative” Life support continues unless complications occur; withdrawal often requires parental assent/consent	Parental participation in care, memory-making, active counseling Emotional and spiritual support Room for attachment and hope, but also for detachment and hopelessness If infant is still within the defined gray zone at 1 week of age, withdrawal of life support is carried out unless parents explicitly ask for continuation of life support

## Data Availability

Not applicable.
